# Assessment of Epstein-Barr virus nucleic acids in gastric but not in breast cancer by next-generation sequencing of pooled Mexican samples

**DOI:** 10.1590/0074-02760150405

**Published:** 2016-03

**Authors:** Ezequiel M Fuentes-Pananá, Violeta Larios-Serrato, Alfonso Méndez-Tenorio, Abigail Morales-Sánchez, Carlos F Arias, Javier Torres

**Affiliations:** 1 Hospital Infantil de México Federico Gómez, Unidad de Investigación en Virología y Cáncer, México, DF, México; 2 Escuela Nacional de Ciencias Biológicas, Unidad Profesional Lázaro Cárdenas, Laboratorio de Biotecnología y Bioinformática Genómica, México, DF, México; 3 Universidad Nacional Autónoma de Mexico, Instituto de Biotecnología, Departamento de Genética del Desarrollo y Fisiología Molecular, Cuernavaca, Morelos, México; 4 Centro Médico Nacional Siglo XXI, Hospital de Pediatría, Unidad de Investigación Médica en Enfermedades Infecciosas y Parasitarias, México, DF, México

**Keywords:** gastric cancer, breast cancer, gastritis, wide genome sequencing, EBV

## Abstract

Gastric (GC) and breast (BrC) cancer are two of the most common and deadly tumours. Different lines of evidence suggest a possible causative role of viral infections for both GC and BrC. Wide genome sequencing (WGS) technologies allow searching for viral agents in tissues of patients with cancer. These technologies have already contributed to establish virus-cancer associations as well as to discovery new tumour viruses. The objective of this study was to document possible associations of viral infection with GC and BrC in Mexican patients. In order to gain idea about cost effective conditions of experimental sequencing, we first carried out an *in silico* simulation of WGS. The next-generation-platform IlluminaGallx was then used to sequence GC and BrC tumour samples. While we did not find viral sequences in tissues from BrC patients, multiple reads matching Epstein-Barr virus (EBV) sequences were found in GC tissues. An end-point polymerase chain reaction confirmed an enrichment of EBV sequences in one of the GC samples sequenced, validating the next-generation sequencing-bioinformatics pipeline.

Globalisation, climate change, urbanisation of wild areas, and other modern conditions of life are contributing to an increased exposure to a number of infectious agents. Concomitantly, worldwide populations are experiencing a demographic transition that has placed cancer as one of the leading causes of death. To date, a number of pathogens, mostly viruses, have been classified as carcinogenic to humans by the International Agency for Research on Cancer. These viruses include: high-risk human papillomavirus (HPV), Epstein-Barr virus (EBV), Kaposi sarcoma herpesvirus, Merkel cell polyomavirus (MCPyV), hepatitis B virus, hepatitis C virus, and human T-lymphotropic virus 1 (Morales-Sánchez & Fuentes-Pananá 2014). Most families of oncogenic viruses are either strict DNA or viruses in which their life cycle has a DNA stage, such as retroviruses, which convert their genomic RNA into proviral DNA that is integrated into the host genome.

It is estimated that human cancers of viral aetiology comprise up to 20% of all tumours, with higher frequencies found in developing countries (Crawford 2005). However, viral agents often defy isolation and recognition by traditional culture or molecular methods. Next-generation sequencing (NGS) technologies are currently used for wide-genome sequencing (WGS) providing high throughput data from single genomes, making them optimal to interrogate for the presence of known and previously unrecognised viral agents. Furthermore, WGS has recently allowed the identification of a novel polyomavirus associated with Merkel cell carcinoma (MCC) (Feng et al. 2008).

Gastric (GC) and breast (BrC) cancer are two of the most common cancers and two of the most important problems in public health now days. GC is the fifth most frequent cancer worldwide and the third cause of death by cancer, whilst BrC is the most frequent cancer in working-age woman (Ferlay et al. 2014). GC is considered primarily of infectious aetiology, with *Helicobacter pylori*infection recognised as the most important risk factor (Crew & Neugut 2006). Colonisation of the gastric mucosa by *H. pylori* triggers a chronic inflammatory response that when unregulated chronically damages the gastric mucosa, generating progressive lesions of increased severity and risk of ending in cancer (Correa et al. 2006). Lesions usually start with a nonatrophic gastritis (NAG), progress to atrophic gastritis, intestinal metaplasia and dysplasia, to finally evolve into GC (Correa et al. 2006). More recently, several lines of evidence also support infection by EBV as an important causative agent for GC (Murphy et al. 2009, Camargo et al. 2011).

Several risk factors have also been described for BrC, including a family and personal history of BrC, increased number of menstrual cycles, reproductive history, hormone therapy, cigarette smoking, and obesity (Kubista 2001). Viral infection has also been suggested as a risk factor for BrC, and mouse mammary tumour virus (MMTV), HPV and EBV are the agents reported as probably associated with BrC (Joshi & Buehring 2012). However, data have been highly variable, with reported infection prevalence ranging from 0-100% and the viral infection-BrC association remains highly controversial.

In this study, we searched for fingerprints of viral infection in pools of GC and BrC tissues using the IlluminaGallx NGS platform. Previously, we implemented an *in silico* NGS simulation assay aimed to find a manageable cost effective pipeline of analysis to interrogate for the presence of viral sequences in cancer samples. We did not find sequences supporting viral participation in breast tumours, whereas multiple reads matching EBV sequences were found in gastric tumours. An end-point polymerase chain reaction (PCR) confirmed EBV sequences in one of the GC samples sequenced ratifying the utility of the bioinformatics pipeline of analysis. The development and implementation of specific and sensitive NGS together with bioinformatics strategies will become critical to dissect the biome associated with cancer and many other diseases of infectious origin.

## SUBJECTS, MATERIALS AND METHODS

*Study population* - Patients with confirmed diagnosis of GC, BrC, and NAG were included in the study. Five patients formed every study group. All patients were recruited in Mexico City, GC and BrC patients from the Oncology Hospital and patients with a NAG diagnosis from the Specialities Hospital, both from Mexican Institute of Social Security (IMSS) at the XXI Century National Medical Center. Tumour and tumour-adjacent tissues were derived from the organ resection. Tumour-adjacent tissues served as controls for specificity of tumour cell infection and these control samples were taken ≥ 2 cm apart from the tumour mass from the same patient in which tumour tissue was obtained. Gastric biopsies were from patients referred to the gastroenterology unit of the Specialities Hospital because of gastric symptoms. A fragment of all tissues was fixed in formaldehyde and embedded in paraffin and a slide was stained with haematoxylin-eosin and analysed by a pathologist to confirm the diagnosis. All tumour tissues included in the study were carcinomas with at least 70% of tumour cells; all GCs included in the study were classified as mixed type (intestinal and diffuse) according to the Lauren’s criteria. All BrC were classified as ductal infiltrating. The BrC molecular classification was the following: three patients were luminal A, one patient was with human epidermal growth factor receptor 2 positive, and one patient was triple negative. In the gastritis samples, we included cases without atrophy or pre-neoplastic lesions with a diagnosis of NAG.

*Sample preparation* - Ten milligrams of each tissue sample were disrupted in a TissueLyser II (Qiagen, Germany) for 20 s and homogenates were subjected to DNA purification with QIAamp DNA mini kit in a QIAcube automated sample processing workstation (Qiagen). Purified DNA was quantified using a spectrophotometer NanoDrop 1000 (Thermo Fisher Scientific, USA) and DNA quality was determined with the 260/280 ratio of absorbance, integrity by electrophoresis in agarose gels, and by PCR of β-actin (670 bp) endogenous gene using primers previously described (Fuentes-Pananá et al. 2004).

*Sample analysis* - DNA from BrC, BrC tumour-adjacent controls, GC, GC tumour-adjacent controls, and NAG was sequenced. Pools of five patients formed every group of study for a total of five pools. We used 1 μg of DNA from each patient for sequencing a total of 5 mg per group. DNA from each group was loaded into separated lanes of a flow cell from a Genome Analyzer IIx (Illumina, USA). Sequencing was performed through 36 cycles of single base pair extensions. Fluorescent images were analysed using the Illumina base calling pipeline v.1.4 to obtain data sequences. The resulting initial sequences of the samples have a length of 36-mer. Those reads obtained for all samples (in FASTQ format) were filtered from undesired sequences using the assembly Perl tools from the Euler-SR program (Chaisson & Pevzner 2008). Then, a collection of programs developed with the Lazarus Free Pascal programming language was used to (i) eliminate sequences of low complexity such as mononucleotide repeats, (ii) trim end-nucleotides that did not fulfill a phred quality value < 30, (iii) change the data file format from FASTQ to FASTA, and (iv) eliminate repeated reads.

*In silico preliminary analysis* - Simulation of Illumina Sequencing was carried out in ART (Huang et al. 2012) with the ART’s parameterised quality profiles and model error specific of the platform. The inputs contained two copies of human genome (GRCh37.p13) plus one of the following options: (i) 100 copies of the HPV 16 genome (NC_001526.2) (Theelen et al. 2010), (ii) 10 copies of the MMTV genome (NC_001503.1) (Morris et al. 1977), or seven copies of the EBV genome (NC_009334.1) (Liu et al. 2011). Emulation generated synthetic Illumina sequencing reads according to different covertures (0.1X, 0.2X, 0.5X and 1X) and two different read sizes (36 mer and 100 mer). Subtraction of poor quality and human sequences, as well as mapping of viral sequences, is described in the next section.

*Pipeline bioinformatics analysis* - This pipeline of analysis was carried out for the *in silico* data first and served as a guidance to set the conditions of the sample sequencing and analysis. Here, the search for reads matching viral genomic sequences was performed according to the method developed by Aleksandar (Kostic et al. 2011). Reads were compared against human sequences using the Bowtie short read aligner v.0.12.7 (Langmead et al. 2009) considering 2, 1, or 0 mismatches, and after a preliminary analyses only 1 mismatch was allowed throughout the final analysis. A human genome databank was created for this comparison, which contained five different genomic databases: three derived from male assemblies hs_ref_GRCh37.p2 (2009), hs_refHuRef (2007), and hs_alt_Celera (2001) (ftp.ncbi.nih.gov/genomes/H_sapiens/), one female assembly (2008) (ftp.1000genomes.ebi.ac.uk), one mitochondrial (2010) (NC_012920.1), plus two transcriptome databases: National Center for Biotechnology Information (NCBI)*Homo sapiens* RNA database (ftp.ncbi.nih.gov/genomes/H_sapiens/RNA/) and Ensembl *Homo sapiens*cDNA database (ftp.ensembl.org). The subtraction of human reads was done with a suite of PERL developed programs. The remaining non-human reads were analysed by BLASTN (v.2.2.28, word size = 9, E-value = 1 x 10^-6^) to 1,520,849 viral sequences [downloaded from NCBI Nucleotide (ncbi.nlm.nih.gov/nucleotide)] using the search term “viruses” [porgn: txid10239] on 1 January 2013. The viral reads obtained were contrasted against a dataset of bacterial, protozoa, and fungi sequences to confirm their authenticity using Bowtie (parameter 1 mismatch). The dataset was a compilation of the following: 3,336 bacteria complete genomes (2012) (ftp://ftp.ncbi.nih.gov/genomes/Bacteria/), 500 human microbiome bacteria (ftp.ncbi.nih.gov/genomes/HUMAN_MICROBIOM/Bacteria/), 716,562 protozoa sequences (NCBI Nucleotide database terms: “apicomplexans” [porgn: txid5794], “amoebozoa” [porgn: xid554915], diplomonadida [porgn: txid5738], “kinetoplastida” [porgn: txid5653], “platyhelminthes” [porgn:__txid6157]), and 522,571 fungi sequences (NCBI nucleotide database terms: “ascomycetes” [porgn: txid4890], “neocallimastigales” [porgn: txid29006], “microsporidians” [porgn: txid6029], “mucorales” [porgn: txid4827], “glomerales” [porgn: txid1028384], “tremellomycetes” [porgn: txid155616]). All generated sequences were deposited in SRA database with the next BioSample accessions 774161 (BrC), 795877 (BrC control), 795890 (GC), 796157 (GC control), and 796243 (gastritis). The analysis pipeline was constructed using the Perl 5.8.1 program. Data processing was carried out in a Mac Pro equipment with a Mac OS X server operating system, a 2x2.66 GHz 6-core Intel Xeon processor, and 32 GB RAM memory.

*Direct search of viral sequences* - To corroborate the lack of hits for viral agents previously documented in samples, 6,782 sequences of HPV [porgn: txid10566], 258 sequences of mouse mammary tumour virus [porgn: txid11901], and 814 sequences of bovine leukaemia virus [porgn: txid11901] were downloaded and matches were directly searched in the BrC and breast nontumour control databases. Sequences were aligned using Bowtie, allowing up to 1 mismatch and without removing any human sequence; the only filters used in this latter search were quality phred, mononucleotides, and repeated sequences of data sets.

*PCR detection of EBV* - DNA samples were subjected to a first PCR with primers LLW1 and LLW2 (Labrecque et al. 1995) which amplify a region within the BamHI W fragment in the EBV genome. The Daudi cell line was used as positive control. The PCR mix (50 μL) contained 200 ng of template DNA, 200 µM of dNTPs mix, 2.5 mM of MgCl_2_, 5 μL of Taq Polymerase buffer 10x with (NH_4_)_2_SO_4_, 200 nM of each primer, and 2.5 U of Taq Polymerase (all from Thermo Fisher Scientific). The PCR reaction was: an initial denaturation step of 5 min at 94ºC and then 30 cycles of 94ºC for 1.5 min, 57ºC for 45 s and 72ºC for 1 min, and a final extension of 72ºC for 7 min. Internal primers to the first PCR amplicon were designed for a nested PCR: LLWint1 5’CTTTGTCCAGATGTCAGGGG3’ and LLWint2 5’GCCTGAGCCTCTACTTTTGG3’. The 50 μL PCR mixture contained 1 μL of the first PCR (1:1000 final dilution), 200 µM of dNTPs mix, 2.5 mM of MgCl_2_, 5 μL of Taq Polymerase buffer 10x with (NH_4_)_2_SO_4_, 400 nM of each primer, and 2.5 U of Taq Polymerase (all from Thermo Fisher Scientific). The reaction was performed with an initial denaturation step at 94ºC for 5 min, followed by 15 cycles of 94ºC for 20 s, 57ºC for 20 s, and 72ºC for 30 s, and a final extension of 72ºC for 7 min.

*Sanger sequencing* - The identity of the EBV positive PCR product was confirmed by sequencing both forward and reverse strands. The PCR product was purified using QIAquick gel extraction kit (Qiagen) according to manufacturer’s instructions, and sequencing of the isolated DNA fragment was carried out in the Biology Institute, National Autonomous University of Mexico. Sequences were compared with the GenBank database using the BLAST program (Altschul et al. 1990).

*Ethics* - The National Commission of Scientific Research and the Ethical Committee on Research of the IMSS approved this project. All patients were informed on the nature of the study and those willing to participate signed a written informed consent prior to specimen collection.

## RESULTS

**Fig. 1:**
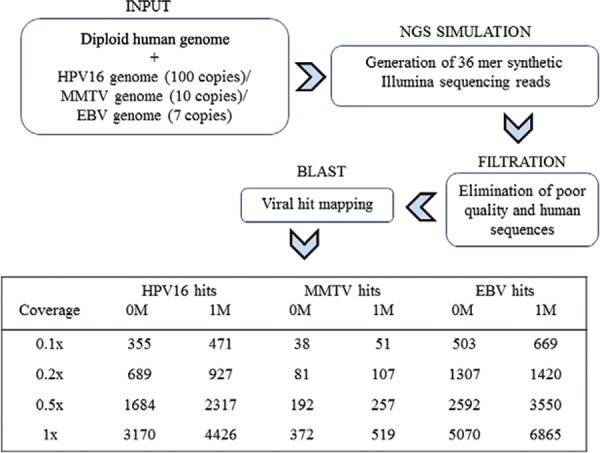
: flowchart of next-generation sequencing (NGS) simulation*.* Illumina sequencing emulation generating 36 or 100 mer (not shown) reads was run in ART (Huang et al. 2012). Different coverages were tested. Several viral hits from human papillomavirus (HPV) 16, mouse mammary tumour virus (MMTV), and Epstein-Barr virus (EBV) were mapped from human-viral inputs mimicking genomic load present in tumours associated to these agents. Viral sequences were screened allowing 0 (0M) or 1 (1M) mismatch. Tens or hundreds of viral reads were found.

*Preliminary simulation analysis* - Looking to implement a bioinformatics pipeline that allowed manageable and cost effective sequencing conditions, we first carried out an NGS simulation analysis. We selected three previously GC and BrC-associated viruses with different genome sizes, ranging from about 8,800 bp (MMTV) to 172,000 bp (EBV). Three different inputs containing human and viral genomes were constructed. The number of human and viral genomes used in every input was adjusted mimicking real infected tumour cells. Thus, 100 copies of HPV 16 genome (Theelen et al. 2010), 10 copies of MMTV genome (Morris et al. 1977), or seven copies of EBV genome (Liu et al. 2011) were used per diploid human genome. Generation of synthetic reads was followed by several rounds of filtration (See Fig. 1 and Subjects, Materials and Methods). Multiple viral reads mapped to filtered data sets. This analysis predicted the minimal coverage to detect viral fingerprints. We also interrogated *in silico* for the most suitable size read, and a 36 mer vs. a 100 mer read size was compared (not shown). This simulation showed that using 36 mer read size was enough to detect viral hits (Fig. 1). This preliminary analysis guided us about adequate conditions of sequencing for viral detection in biological samples.

*NGS in biological samples* - Looking to reduce analysis cost, pools of samples were sequenced based on conditions founded in the preliminary analysis. Our models of study were GC and BrC since multiple lines of evidence support an infectious aetiology for both types of cancer. Also, since most families of tumour viruses can be traced by DNA, DNA samples were sequenced. Looking to strengthen the capacity to find possible viral sequences, only tumour tissues containing at least 70% of tumour cells were included. Tumour-adjacent tissues isolated from the same cancer patients and located at least 2 cm apart from the tumour mass served as controls for specificity of tumour cell infection, while biopsies of patients with NAG addressed the possible viral participation in early inflammatory precursor lesions.

The pipeline analysis of the data generated by the Illumina GAIIx sequencing and the percentage of non-human reads remaining after each level of filtration are shown inFig. 2. Ten to 20% of sequences were eliminated on the bases of quality and composition, 76-88% reads preferentially matched human sequences and were also eliminated, to end with an average of 2.39% non-human reads for all tissues sequenced. Table I shows the number of initial and final non-human reads after all filtration steps. Human sequences were a compendium of seven different databases, which allowed a more stringent tool for filtration and a better selection of sequences with more distant similarities to human genomes. The database of human sequences can be found freely at NCBI and European Molecular Biology Laboratory, and all human sequences found in pools of sequenced tissues at drive.google.com/folderview?id=0B3AZ9N8M5ZMfVGtpbUFtQ1JBbE0&usp=sharing for readers use.

**TABLE I:** Number of reads

Tissue	Total reads	Non-human reads
BrC	15,600,870	269,740
BrC control	30,100,894	513,073
GC	29,213,391	770,760
GC control	27,296,544	507,667
Gastritis	14,421,599	329,850

BrC: breast cancer; GC: gastric cancer.

**Fig. 2:**
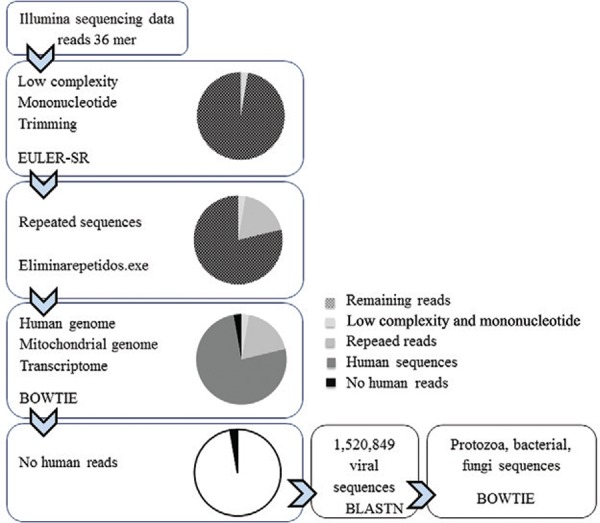
: pipeline of analysis of deep sequencing data. next-generation sequencing crude data were enriched of viral sequences by subtracting unwanted reads as follows: (i) filtering low-quality reads by EULER-SR, (ii) repeated sequences by Eliminarepetidos.exe, (iii) human sequences aligning by BOWTIE, (iv) other nonviral reads by aligning with BLASTN against publicly available sequence databases (protozoan, bacterial, and fungi sequences). Pie charts illustrate the subtracted and remaining non-human reads. For a more in deep explanation see materials and methods.

The number of non-human reads obtained constituted a set of manageable information for more robust analysis; such reads were blasted against different microbial databases, including a database of 1,520,849 viral sequences. The viral database was based in available sequences deposited in the NCBI, and it is also freely available (drive.google.com/folderview?id=0B3AZ9N8M5ZMfYjJCT0xlNzZSdkU&usp=sharing).

Different types of viral hits were found, most of them were of no interest since they were not from members of a family of tumour viruses or because of they were present across all tumour and control tissues. For instance, several hits matched sequences present in phages or vectors that are commonly used as tools for molecular biology research: eight-18 hits matching enterobacteria phage sequences were found across all sequenced tissues, one-two hits matching baculovirus sequences were also found across all tissues, and one hit matching SAdV-40 was found in BrC tissue. The origin of this genetic material most likely comes from contamination with enzymes and reagents used to process tissues. Two hits were found matching the Torque teno virus, which is commonly found in cells of the immune system: one in BrC tumour-adjacent control and one in GC. Slightly more interesting was to find hits matching retroviral sequences; although, those hits showed higher similarity with human endogenous retrovirus H and K and less to oncogenic MMTV, which has been linked to BrC. Furthermore, these retroviral hits were found in all tissues sequenced as shown in Table II.

**TABLE II:** Viral hits

Virus	BrC	BrC control	GC	GC control	Gastritis
EBV (HHV4)	0	0	10	1	2
HHV6	16	45	0	0	0
HHV7	0	1	1	0	4
CMV (HHV5)	0	0	1	1	0
Other herpesviruses	1	0	4	1	0
HERVS	10	6	7	7	5

BrC: breast cancer; CMV: citomegalovirus; EBV: Epstein-Barr virus; GC: gastric cancer; HERVS: human endogenous retrovirus; HHV: human herpesvirus.

Several hits matching members of the Herpesviridae family were found: six against human herpesvirus type 7 (HHV7), one in BrC tumour-adjacent, one in GC, and four in NAG; two against citomegalovirus, one in GC, and one in GC tumour-adjacent; six against herpes simplex virus type 1 (HSV1), one in BrC, one in GC tumour-adjacent, and four in GC. These HSV1 sequences also exhibited high similarity (> 90%) to other members of the Alphaherpesvirinae subfamily, to gallid herpesvirus 2 (infects birds), angullid herpesvirus (infects eels), and cyprinid and Koi herpesvirus (both infect fish).

Hits matching HHV type 6 (HHV6) and EBV sequences exhibited tissue specificity; HHV6 had 61 hits in breast and zero in gastric tissue. However, there was not specificity of infection for the tumour sample (16 hits in BrC and 45 in BrC tumour-adjacent control tissue); therefore, this result does not support a direct etiological role for HHV6 in BrC. On the other hand, EBV had 10 hits in GC, while only one in GC tumour-adjacent tissue and two in NAG. Table III shows the identity of the gene targets of the EBV hits. One hit overlaps to two overlapping coding sequences: EBNA-2 and BYRF1.

**TABLE III:** Identity of Epstein-Barr virus hits

Tissue	Read	Target gene
GC	TCACGGCATCTGGGGTGACCGGGGCCATCGGGTTT	BFLF2
GC	GATGCCCTCCAGGTCAAAGACGTTGGAGGCACGCT	BALF4
GC	AGGGAGTCACGTAGGCACTAGACTCTTCATGTGAG	BART miRNA
GC	GGTTGAGGTGGTAAAGACGTGGGCCGTGGTCAGAT	BGLF2
GC	GTTACATGGGGGACAAACATATCATCTAATTGTTG	EBNA-2/BYRF1
GC	ACCGGCGTGCGAGGAGCAGCATGCAGGCTCGGGCG	BPLF1
GC	GGGGGCGGCCGGCCAGGAAGCTCTTGACCAGGTAG	BPLF1
GC	CTGACTTTCATGAAATCCCTGACCTCGATGACTCC	BRRF2
GC	CCCTACTTGGGAGAGTCCGGCAAGGCCAGAGACAC	BMRF2
GC	TCCGGGGTAAGCTTCGGCCATGGCCGGAGCTCGTC	LF1
GC control	CTTTGCGGCCCCGACTATCGACCCTGCATTTGCGA	BPLF1
Gastritis	TCCTCGGAGCCAGCCCCCGGGGTTGGTTCTGCCCC	EBNA-LP
Gastritis	AAGATGCGAGTTTGCAATGTCCCCCGCCTCATCAA	BCRF1

GC: gastric cancer.

No evidence of viral participation in BrC was found even though several lines of evidence point out for a possible participation of HPV, MMTV, bovine leukaemia virus, and EBV in the aetiology of this cancer. To further support our previous observation, direct searches of these viruses against the tumour and control tissues sequences were performed with similar results; no hits for these viruses were found. In contrast, when EBV sequences were contrasted against unfiltered sequences from all tissues, 103 hits were found in GC, 12 hits in adjacent control, and 29 hits in gastritis samples.

Although, the main goal of this study was to analyse the virome of BrC and GC, but since *H. pylori* infection is recognised as the main risk factor to develop NAG and GC, a blast of the non-human reads found in gastric lesions was performed against a database of 3,336 full bacterial genomes. These data is shown in Supplementary Figure. Hits mapping to the *Helicobacter* genus were found in 2%, 12%, and 36% of all bacterial reads in GC, GC adjacent tissue control and NAG samples, respectively. *Helicobacter* was the most abundant genera found in gastritis, while *Paracoccus* and*Propionobacterium* were the most represented in GC and GC controls. These data is congruent with the literature documenting that *H. pylori* tents to be absent of tumour tissues, while it is highly abundant in early gastric lesion (Kokkola et al. 2003, Camorlinga-Ponce et al. 2008).

**Fig. 3:**
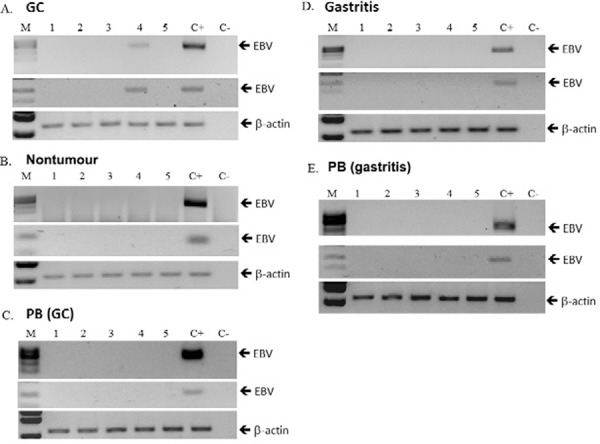
: detection of Epstein-Barr virus (EBV) by polymerase chain reaction (PCR). A: one of five gastric cancer (GC) samples was positive (sample four) to EBV; by first round PCR (upper panel) and nested PCR (middle panel); B: nontumour control tissues (C) and peripheral blood (PB) from the same GC patients were negative to EBV by both PCRs; D: none of the gastritis samples (E) nor PB of these patients were positive to EBV by any PCR. DNA from Daudi cells was used as positive control (C+). A reaction without DNA was used as negative control (C-); M: molecular weight marker. Lower panels shows the PCR of loading and DNA integrity control, the endogenous gene β-actin.

*EBV detection by PCR* - To confirm the presence of EBV in GC tumours and validate the results from the WGS, a PCR test was implemented using primers LLW1 and LLW2 (Labrecque et al. 1995) in a reaction set to detect ≥ 40,000 viral genomes (Martínez-López et al. 2014). Tissues from the five GC tumours and their counterpart tumour-adjacent controls and the five NAG samples were individually tested by the PCR. Furthermore, since EBV usually resides in B cells in a low number of infected cells, mononuclear cells isolated from peripheral blood of GC and NAG patients were also included in the analysis. Fig. 3 shows the result of the PCR test; one of the GC tissues was found positive for EBV sequences, while the other four patients were negative. The patient positive by the PCR test was negative in the tumour-adjacent control tissue and peripheral mononuclear cells, supporting an enrichment of EBV infected cells in the tumour tissue. None of the NAG patients were positive, in agreement with the NGS data. EBV sequences were confirmed with a more sensitive nested PCR (detects ≥ 1,500 viral particles) (Martínez-López et al. 2014) and by Sanger sequencing of the first positive PCR-amplicon.

Taken together, these data highlight the importance of NGS technologies as a powerful and manageable tool to interrogate cancer tissues for the presence of viral sequences looking to better understand the aetiology of the disease.

## DISCUSSION

NGS technologies have opened new perspectives for viral research and diagnostic in multiple human and veterinary diseases. In recent years, this technology has allowed the identification and characterisation of new viruses, such as the Bundiubugyo virus, a virus related to Ebola and responsible for severe haemorrhagic fevers in humans (Towner et al. 2008), and an arenovirus closely related to lymphocytic choriomeningitis viruses (Kim et al. 2011), associated with fatal post-transplant disease. MCC was a long suspected cancer of infectious aetiology because it develops preferentially in immunosuppressed individuals. However, the identity of the causative agent remained elusive until high throughput sequencing and transcriptome subtraction methodologies allowed the identification of a novel polyomavirus in MCC samples (Feng et al. 2008). Now, it has been firmly documented that MCPyV is responsible for up to 100% of MCC (Agelli et al. 2010).

Here, we used a similar approach to interrogate for the presence of viral sequences in BrC and GC tissues, two of the most common cancers. However, considering that NGS-based methodologies result in an enormous set of data difficult for processing and interpretation, we implemented some strategies to reduce both cost and time of analysis to the experimental and bioinformatics approach. DNA samples were pooled (with DNA from 5 patients in every lane of the sequencer), thus, we reduced the cost of massive sequencing. Additionally, we carried out an *in silico*preliminary analysis simulating several NGS conditions according to the reported viral genome copies found in cancer. Through this analysis, we selected an affordable but sufficient coverage to detect viral sequences.

A database of multiple human sequences was constructed and used to digitally subtract candidate sequences of viral origin, especially those matching members of viral families with oncogenic characteristics. This strategy leaded us to find HHV6 sequences associated with breast tissue and EBV with gastric tumour samples. GC has been extensively associated with *H. pylori* infection and more recently with EBV, and we found evidence of both pathogens in gastric samples. Since EBV is a widespread pathogen and NGS could detect low levels of contaminant virus, sequencing data were confirmed *via* two PCR tests of increased sensitivity. EBV was detected in the tumour, but not in tumour-adjacent tissue or peripheral mononuclear cells of one GC patient. EBV usually resides in B-cells in frequencies estimated between one-20 cells per million (Rickinson & Kieff 2007), a frequency of infection that was under the limit of detection of either PCR (Ryan et al. 2009, Martínez-López et al. 2014). The enrichment of EBV infection observed in tumour tissue is in agreement with the known direct oncogenic mechanism of EBV through expression of viral oncogenes within the transformed cell. Multiple lines of evidence now support a role for EBV in GC and a recent meta-analysis reports a 10% world-wide prevalence of EBV associated GCs (Murphy et al. 2009,Camargo et al. 2011). We found a similar frequency of EBV infection in Mexican GC samples (Martínez-López et al. 2014). In EBV associated lymphomas, the number of viral copies has been estimated in 50 viral episomes per tumour cell (Gulley et al. 1994), while seven viral copies were found in an EBV associated nasopharyngeal carcinoma (Liu et al. 2011), which is within the limits of the PCR test used here. It is possible that gastric carcinoma more closely resembles NPC, which highlights the power of the NGS even in pools of tissues from different patients.

Like EBV, HHV6 also belongs to the Herpesviridae family and it is also a highly common infection. HHV6 is the causative agent of roseola infantum and together with HHV7 are classified as the human roseoloviruses. HHV6 has been associated with the nodular sclerosis form of Hodgkin’s lymphoma (Siddon et al. 2012), although its role in other tumours is unknown. Our NGS result does not support an oncogenic role for HHV6 in BrC, since viral sequences were found in both BrC and tumour-adjacent tissues. There are evidences for a modulatory role in tumour development for HHV6; for instance, HHV6-induced secretion of interleukin-2 causes T-cell leukaemias to progress more rapidly (Ojima et al. 2005). Contrary to EBV, HHV6 presents a wide range of tropism, infecting all types of immune cells, neurons, and fibroblasts (Yamanishi et al. 2007). HHV6 increased infection/reactivation often occurs in immunosuppressed individuals. Since we observed HHV6 infection specific to BrC but no GC patients, cancer induced immunosuppression would not explain the enrichment of HHV6 in breast tissue; although our observation is more in line with HHV6 infecting nontumour cells. HHV6 infection may promote inflammation and thus indirectly participate in tumour growth as it has been recently shown for b and g herpesviruses (Abate et al. 2015, Pandya et al. 2015). Still, our data is a preliminary observation that needs to be addressed in future studies. In any case, our work also shows the importance of studying in parallel tumour and tumour-adjacent tissues to be able to identify the specificity of the viral infection and the plausibility of its association with cancer development.

Different studies also support an association of HPV with GC and HPV, EBV, and MMTV with BrC. However, reports have been highly variable, with evidence in favour or against, and the issue remains controversial (Sasco et al. 1993, Wang et al. 1995,Zapata-Benavides et al. 2007, Park et al. 2011). Although, some studies support up to 80% of BrC with an infectious aetiology (Joshi et al. 2009) and even infection by multiple viruses (Glenn et al. 2012), we did not find evidence of these associations. According with our results, Tang et al. (2013) analysed transcriptome sequencing reads from 810 BrC tumours finding no support for viral aetiology.

A recent study by Widschwendter et al. (2004)found that secondary BrC after invasive cervical cancer is importantly associated with the presence of HPV DNA, suggesting viral spread and a possible etiological role for HPV. Widschwendter et al. (2004)highlight the importance of knowing the previous infection history of the patients. We do not know the EBV or HPV infection status of the patients. However, both viruses are highly prevalent worldwide. In Mexico, tissue-specific HPV detection has been performed for research purposes in patients with cervical, anal, oral, and other HPV-related cancers, as well as in individuals with human immunodeficiency virus infection (Berumen et al. 2001, Volkow et al. 2001, Anaya-Saavedra et al. 2008, Méndez-Martínez et al. 2014). In cervical cancer, HPV has been found in close to 100% of samples supporting the high prevalence of infection in the adult population. Similarly, EBV is a ubiquitous agent infecting approximately 95% of the adult population worldwide (Henle et al. 1969). In Mexico, there are not population-based studies of EBV seroprevalence, but in our group we analysed Mexican patients with diagnoses of NAG, GC, and premalignant lesions, finding 94.2% of EBV positive cases (Cárdenas-Mondragón et al. 2015). Therefore, it is possible that in this study all patients were infected with HPV and EBV.

We observed a significant difference in the sensitivity of emulated vs. real sequencing. For example, in the case of EBV screening, the number of viral hits found *in silico* was of several hundred while only 10 hits were found in the experimental sequencing (equivalent to 50 hits if we consider that the sequenced sample was a pool of 5 genomes). That means than between the number of synthetic and real reads there is roughly a log difference. Thus, virus whose genomes is short and may be present as low copy number as MMTV could have been lost in experimental sequencing. Therefore, although *in silico*preliminary analyses can help to predict the scope of experimental strategies, we recommend considering this difference in future studies. Notably, we found no reads for MMTV and HPV and we confirmed the absence of MMTV in BrC by PCR (Morales-Sánchez et al. 2013). In this study, we developed and *in silico* pipeline of analysis of viral sequences in tumour samples, which was then used to sequence BrC and GC tumours samples, finding evidence of EBV in GC, a result that validated the sequencing and analysis strategy. An etiological role for EBV in GC is now accepted. Using deep sequencing, The Cancer Genome Atlas (TCGA) research network has identified an EBV+ molecular subgroup of gastric adenocarcinoma in a large set of GC specimens. The EBV+ GC is tightly clustered by a characteristic viral gene expression program abundant in EBV BART microRNAs expression, CpG island hypermetilation, PI3K mutations, and PD-L1/2 overexpression (CGARN 2014). Our study supports that EBV also associates with GC in Mexican population, which was not included in TCGA study. NGS technologies together with rigorous analysis by web-based bioinformatics pipelines will become an invaluable tool to identify causative associations in multiple diseases. The design of optimal strategies will help to reduce biological, economical, and computational resources and, importantly, it may facilitate to translate sequencing technologies into clinical application in countries with limited resources.
